# High-throughput optimisation of light-driven microalgae biotechnologies

**DOI:** 10.1038/s41598-018-29954-x

**Published:** 2018-08-03

**Authors:** Shwetha Sivakaminathan, Ben Hankamer, Juliane Wolf, Jennifer Yarnold

**Affiliations:** The University of Queensland, Institute for Molecular Bioscience, 306 Carmody Road, St Lucia, Australia

## Abstract

Microalgae biotechnologies are rapidly developing into new commercial settings. Several high value products already exist on the market, and systems development is focused on cost reduction to open up future economic opportunities for *food, fuel* and *freshwater* production. Light is a key environmental driver for photosynthesis and optimising light capture is therefore critical for low cost, high efficiency systems. Here a novel high-throughput screen that simulates fluctuating light regimes in mass cultures is presented. The data was used to model photosynthetic efficiency (PE_µ_, mol photon^−1^ m^2^) and chlorophyll fluorescence of two green algae, *Chlamydomonas reinhardtii* and *Chlorella* sp. Response surface methodology defined the effect of three key variables: *density factor* (D_f_, ‘culture density’), *cycle time* (t_c_, ‘mixing rate’), and *maximum incident irradiance* (I_max_). Both species exhibited a large rise in PE_µ_ with decreasing I_max_ and a minimal effect of t_c_ (between 3–20 s). However, the optimal D_f_ of 0.4 for *Chlamydomonas* and 0.8 for *Chlorella* suggested strong preferences for dilute and dense cultures respectively. *Chlorella* had a two-fold higher optimised PE_µ_ than *Chlamydomonas*, despite its higher light sensitivity. These results demonstrate species-specific light preferences within the green algae clade. Our high-throughput screen enables rapid strain selection and process optimisation.

## Introduction

Green algae are oxygenic photosynthetic organisms which, like higher plants and cyanobacteria, have evolved over 3 billion years to tap into the huge energy resource of the sun. This energy is used to fix CO_2_, releasing O_2_ as a by-product and producing biomass rich in proteins, lipids, starch, bioactive compounds and phytonutrients. Consequently, single celled green algae (microalgae) are increasingly being integrated into industrial production systems to realise solar driven biotechnologies. Microalgae technologies are already being exploited commercially to produce high value commodities (e.g. functional foods, feeds, protein therapeutics and chemicals)^1–3^ and the knowledge gained is driving down production costs toward the levels required to expand low value market opportunities including fuels and fertilisers as well as ecosystem services (e.g. water treatment and CO_2_ sequestration)^4–6^. The first step of all solar driven microalgae processes is light capture and conversion to chemical energy (ATP, NADPH), and the optimisation of this step is therefore essential to develop high-efficiency economic solutions^7–9^. In outdoor mass cultures, the light reaching the surface of the pond or bioreactor is highly variable over the day, ranging from light limiting during early/late hours of the day or periods of high cloud cover, to photo-inhibiting conditions (up to 2,000 µmol m^−2^ s^−1^) during mid-day in locations receiving high solar radiation. Within the culture itself, cells are exposed to high light gradients as they cycle from the illuminated surface (often inhibitory light levels) to deep within the culture (light limiting or dark conditions). This fluctuating light regime within the mass culture is governed by the optical properties of the culture (based on cell size, cell number and pigment content) while the frequency with which cells cycle between the light and dark zones is regulated by mixing rate as well as the photobioreactor geometry which influences the light distribution through the optical pathlength and the surface to volume ratio. The relatively rapid light fluctuations within the culture affect the photo-regulatory response, while the relatively slow environmental light fluxes affect photoacclimation, both leading to changes in the overall productivity of the culture^10–12^.

Defining and optimising the effects and interactions of the variables that govern a given light regime is a challenge that requires comparatively large experimental datasets which can be laborious and expensive to obtain using traditional pilot- or even laboratory-scale bioreactors. The high-throughput light screen method presented here has been designed to simulate light regimes encountered in mass cultured photobioreactors under ‘typical’ outdoor production conditions to enable process optimisation, model guided system design, species selection and a better extrapolation of laboratory results to field trials.

The light screen collected data from LED illuminated microwells, and Response Surface Methodology was employed to predictively model photosynthetic efficiency (PE_µ_), to define both main effects and the pair-wise interactions between the light factors that govern it and to identify the conditions that yield optimum productivity. As fluctuating light can effect photoregulation and photoacclimation, we also investigated some of these underlying mechanisms to assess the extent of their effect on PE_µ_.

**Figure 1:**
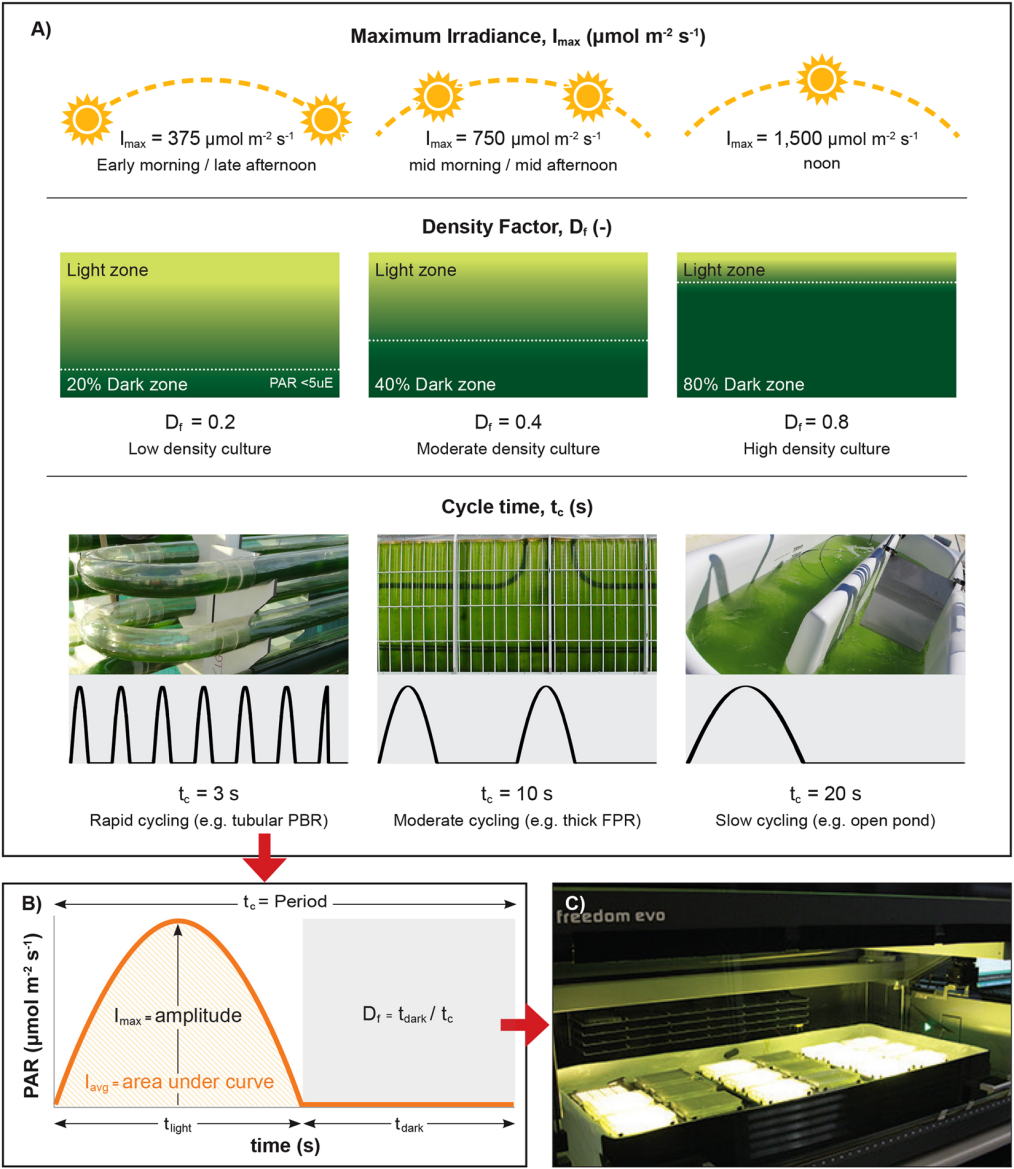
Experimental design for high-throughput light simulations of cells cycling in outdoor microalgae mass cultures. (**A**) Depicts the 3 factors that affect the light regime experienced by cells cycling in mass cultures: I_max_, D_f_ and t_c_, and the levels used for the full factorial experimental design which are based on ‘typical’ outdoor conditions. (**B**) Each combination of light factors was programmed by changing the light intensity of the LEDs over the cycle time, assuming cell cycling occurs in a sinusoidal trajectory. Here, I_max_, is the amplitude of the sine, simulating the maximum irradiance that a cell would receive when at the ‘surface’ of a mass culture, D_f_, is the proportion of time that PAR is below 5 µmol m^−2^ s^−1^ in one period; this simulates the fraction of time that a cell spends in the dark, depending on the culture density, and t_c_ is the period of one sine wave, that simulates the time required for a cell to cycle through the reactor. I_avg_ is the integration of light received, simulating the average irradiance or light dose received the by cell. Here t_light_ and t_dark_ are the time cells receive PAR (>5 µmol m^−2^ s^−1^) and no PAR (<5 µmol m^−2^ s^−1^) respectively. (**C**) The programmed LEDs form part of an 18-plate microwell robotic system. *Chlamydomonas* and *Chlorella* were incubated in 96-well plates placed on LED arrays with one LED per microwell and one unique light regime per plate. All light regimes occurred over a photoperiod of 16 h day^−1^ and a dark period of 8 h day^−1^.

A full factorial experimental design was employed, with quadratic models fitted to the data to measure the PE_µ_ in response to variations of three key factors that govern the light regime to which cells in mass culture are exposed: density factor (‘D_f_’, -), defined as the proportion of the time that cells are in the dark zone (t_dark_, s) compared with the total time in both light (t_light_, s) and dark zones; *cycle time* (‘t_c_’, s), which is defined by the mixing rate, or the total time of a cell’s fluctuation between light and dark zones for one cycle along the culture depth; and maximum irradiance (I_max_, µmol photons m^−2^ s^−1^) defined as the irradiance entering the photobioreactor at the illuminated surface (Fig. 1A). Dark was defined as <5 μmol PAR at which respiration typically exceeds photosynthesis (the compensation point)^13,14^. The three factors (D_f,_ t_c,_ I_max_) affect the average irradiance (I_avg_), which is the integration of light experienced by the cells over the entire light cycle (Fig. 1B). Our miniaturised and automated screen enables the analysis of the interactions between the three light-dependent factors and generates a strain-specific model that can be used to optimise production conditions or predict productivities for different production scenarios.

This empirical model is an alternative approach to traditional models based on photosynthetic irradiance (P–I) curves. It only requires knowledge of the density factor, incident irradiance and mixing rate. The D_f_ for a given species and reactor geometry can be easily found (indoor or outdoor) for a given incident irradiance by measuring the depth of culture at the point where light is reduced to <5 µmol m^−2^ s^−1^ (i.e. start of the “dark zone”) and calculating the ratio of this depth to the total culture depth (usually fixed). This can be correlated to a range of optical densities (or biomass dry weights) to provide a simple method to establish what D_f_ a reactor will have at a known culture density, pathlength and incident irradiance. Since D_f_ has been determined as a critical factor in this and other studies, we believe that this is another useful modelling tool for process design.

Two biotechnologically relevant microalgae strains were analysed in this study: *Chlamydomonas reinhardtii* (*Chlamydomonas*), the model alga most used in photosynthetic studies^15,16^ and for heterologous protein expression^17,18^, and a strain of *Chlorella* sp, 11_H5 (*Chlorella*) isolated in Australia which was found to have high biomass productivity at laboratory and pilot scale^19,20^*. Chlamydomonas* (originally isolated from soil)^21^ has successfully transitioned from land to water in laboratory conditions, arguably owing to its robust and evolved photosynthetic machinery that protects it from oxidative stress and changing environmental conditions^22^. Hence, understanding the interplay between photosynthetic regulation, photoacclimation and its effect on growth and biomass productivity would determine the feasibility of delivering functional microalgae biotechnologies. This paper presents a high-throughput miniaturised light optimisation screen (allowing up to 18 different combinations of light regime and up to 1,728 conditions), designed to identify species-specific illumination conditions that maximise photosynthetic efficiency and productivity to fast track systems optimisation.

## Results

### High-throughput screen (HTS) of simulated light regimes in mass cultures

To analyse the effects of varying levels of I_max_, D_f_ and t_c_ (Fig. 1B) on the PE_µ_ of microalgae, light simulations were performed on dilute 150 μl microwell cultures (5 mm pathlength)^23^, each illuminated using individual LEDs (Fig. 1C). The intensity of photosynthetically active radiation (400–700 nm, PAR) emitted by the LEDs was programmed (Arduino® integrated circuit and controller) to mimic a sinusoidal trajectory of a cell cycling in a one-dimensionally illuminated culture (i.e. an open pond) between the illuminated surface and the dark zone (Fig. 1B)^10^. In this way, the light regime encountered by the incubated cells in each well was a function of the LED’s illumination profile, thereby allowing tight control of the levels of each factor (I_max_, D_f_ and t_c_), (Fig. 1A). A robotic arm was programmed to take the plates to a reader at determined time intervals where rapid measurements of optical density and fluorescence can be taken. Here, two strains were analysed for the initial HTS light simulations, however, this method can rapidly be used to model up to 32 strains run in triplicate in one experiment.

Figure 1A depicts the three levels of each factor (I_max_, D_f_, t_c_) and the real-world phenomena they represent based on information from literature^24–26^ and on experimental data^27–29^. A low (0.2) or high (0.8) D_f_ represents a low or high cell/biomass density respectively (e.g. dilute cultures at the beginning of cultivation *versus* dense cultures at harvest in a batch production regime). The system is able to analyse any range between 10 ms fluctuations to constant light. The cycle time of 3–20 s represents typical ‘mixing’ cell cycle rates through the optical pathlength of photobioreactors, where a t_c_ of 3, 10, and 20 s represents rapid, moderate or slow mixing, as might occur in a tubular PBR, thick flat panel PBR and open pond respectively. The t_c_ is influenced by mixing and/or sparging rates, reactor pathlength, or a combination of the two, which can vary for individual reactors depending on cultivation regime. The I_max_ values represent the incident solar radiation in the early morning and late afternoon (375 µmol m^−2^ s^−1^), mid-morning and -afternoon (750 µmol m^−2^ s^−1^), and noon (1500 µmol m^−2^ s^−1^) respectively. I_max_ values are based on the average annual solar radiation levels for Brisbane, Australia^30,31^, and are representative of other high solar regions that are suitable for outdoor microalgae production. The simulation of these three factors at three levels each via programmed changes in LED light flux over time are depicted in Fig. 1B. This approach provided a complete factorial design (3^3^) of 27 combinations for model fitting of the main response variable, PE_µ_ (Table 1) and underlying responses at the level of PSII (Table 2).

**Table 1:** PE_µ_ of *Chlamydomonas* and *Chlorella* under the experimental matrix of light regimes.

I_max_		D_f_		t_c_		I_avg_	PE_µ_ (mol photon^−1^ m^2^)	
*Actual (µmol m* ^−2^ *s* ^−1^ *)*	*Coded*	*Actual (*−*)*	*Coded*	*Actual (s)*	*Coded*	*(mol m* ^−2^ *h* ^−1^ *)*	*Chlamydomonas*	*Chlorella*
375	−1	0.2	−1	3	−1.73	0.619	0.118 ± 0.0030	0.136 ± 0.017
				10	0		0.099 ± 0.0093	0.142 ± 0.018
				20	1		0.107 ± 0.0031	0.151 ± 0.026
		0.4	0	3	−1.73	0.490	0.174 ± 0.0070	0.183 ± 0.012
				10	0		0.133 ± 0.0079	0.149 ± 0.001
				20	1		0.094 ± 0.0070	0.132 ± 0.018
		0.6*	—	3	−1.73	0.367	0.088 ± 0.0066	0.176 ± 0.007
				10	0		0.099 ± 0.0010	0.167 ± 0.011
				20	1		0.084 ± 0.0100	0.149 ± 0.007
		0.8	1	3	−1.73	0.18	0.040 ± 0.0028	0.277 ± 0.022
				10	0		0.048 ± 0.0000	0.197 ± 0.014
				20	1		0.047 ± 0.0107	0.159 ± 0.006
750	0	0.2	−1	3	−1.73	1.242	0.078 ± 0.0037	0.039 ± 0.003
				10	0		0.063 ± 0.0013	0.054 ± 0.002
				20	1		0.053 ± 0.0022	0.076 ± 0.001
		0.4	0	*3*	−1.73	0.979	0.060 ± 0.0121	0.087 ± 0.004
				*10*	*0*		0.061 ± 0.0040	0.087 ± 0.006
				*20*	*1*		0.049 ± 0.0020	0.095 ± 0.008
		0.6*	*—*	3	−*1.73*	*0.738*	*0.079* ± *0.0030*	*0.099* ± *0.005*
				10	0		*0.061* ± *0.0016*	*0.082* ± *0.006*
				20	1		*0.049* ± *0.0030*	*0.182* ± *0.00*3
		0.8	1	*3*	−1.73	0.360	0.063 ± 0.0073	0.134 ± 0.012
				*10*	*0*		0.046 ± 0.0023	0.072 ± 0.022
				*20*	*1*		0.020 ± 0.0027	0.097 ± 0.008
1500	1	0.2	−1	3	−1.73	2.480	0.051 ± 0.0027	0.021 ± 0.0004
				10	0		0.067 ± 0.0109	0.025 ± 0.002
				20	1		0.049 ± 0.0021	0.047 ± 0.006
		0.4	0	3	−1.73	1.958	0.053 ± 0.0021	0.037 ± 0.004
				10	0		0.052 ± 0.0035	0.055 ± 0.001
				20	1		0.045 ± 0.0026	0.072 ± 0.011
		0.6*	*—*	*3*	−*1.73*	*1.472*	*0.050* ± *0.0138*	*0.067* ± *0.001*
				*10*	*0*		*0.041* ± *0.0074*	*0.057* ± *0.006*
				*20*	*1*		*0.030* ± *0.0080*	*0.092* ± *0.003*
		0.8	1	3	−1.73	0.713	0.051 ± 0.0053	0.072 ± 0.001
				10	0		0.031 ± 0.0088	0.043 ± 0.006
				20	1		0.030 ± 0.0170	0.043 ± 0.007

All data are the mean of 3 replicates ± standard deviation. *Indicates data used for model validation. ‘Coded’ refers to the normalised values used for the quadratic model (Equation 2).

**Table 2:** Comparison of the factor coefficients of the quadratic model obtained from analysis of variance (ANOVA) for A) PE_µ_, B) Φ_PSII_ and C) F_v_/F_m_ parameters for *Chlamydomonas* and *Chlorella*. *Represents significant effects at p-value < 0.05. *n* = 3 (PE_µ_), *n* = 2 (ΦPSII & F_v_/F_m_).

	Coefficients from the quadratic non-linear model					
	PE_µ_ (10^−3^)		Φ_PSII_ (10^−3^)		F_v_/F_m_ (10^−3^)	
	*Chlamydomonas*	*Chlorella*	*Chlamydomonas*	*Chlorella*	*Chlamydomonas*	*Chlorella*
D_f_	−21.0^*^	20.5^*^	−35.7^*^	−8.1^*^	16.4^*^	16.6^*^
I_max_	−20.0^*^	−61.2^*^	−3.2	−4.4	22.1^*^	−54.2^*^
t_c_	−6.6	−5.5^*^	−3.3	−2.0	−0.9	−6.8^*^
D_f_ − I_max_	16.0^*^	−10.3^*^	−29.6^*^	−6.8^*^	−6.1	9.7^*^
D_f_ − t_c_	−1.1	−14.7^*^	0.8	0.9	−3.6	3.9
I_max_ − t_c_	3.2	−9.5^*^	−5.0^*^	3.9	3.0	−6.5^*^
D_f_^2^	−24.6^*^	2.4	−26.8^*^	−4.7	10.4	1.8
I_max_^2^	14.2^*^	28.0^*^	19.1^*^	−4.9	31.8^*^	1.7
t_c_^2^	1.0	2.8	−0.2	0.6	1.7	−3.8
Intercept	67.6	71.1	236.5	194.3	655.6	647.1
R^2^	0.67	0.93	0.89	0.44	0.74	0.91

**Figure 2:**
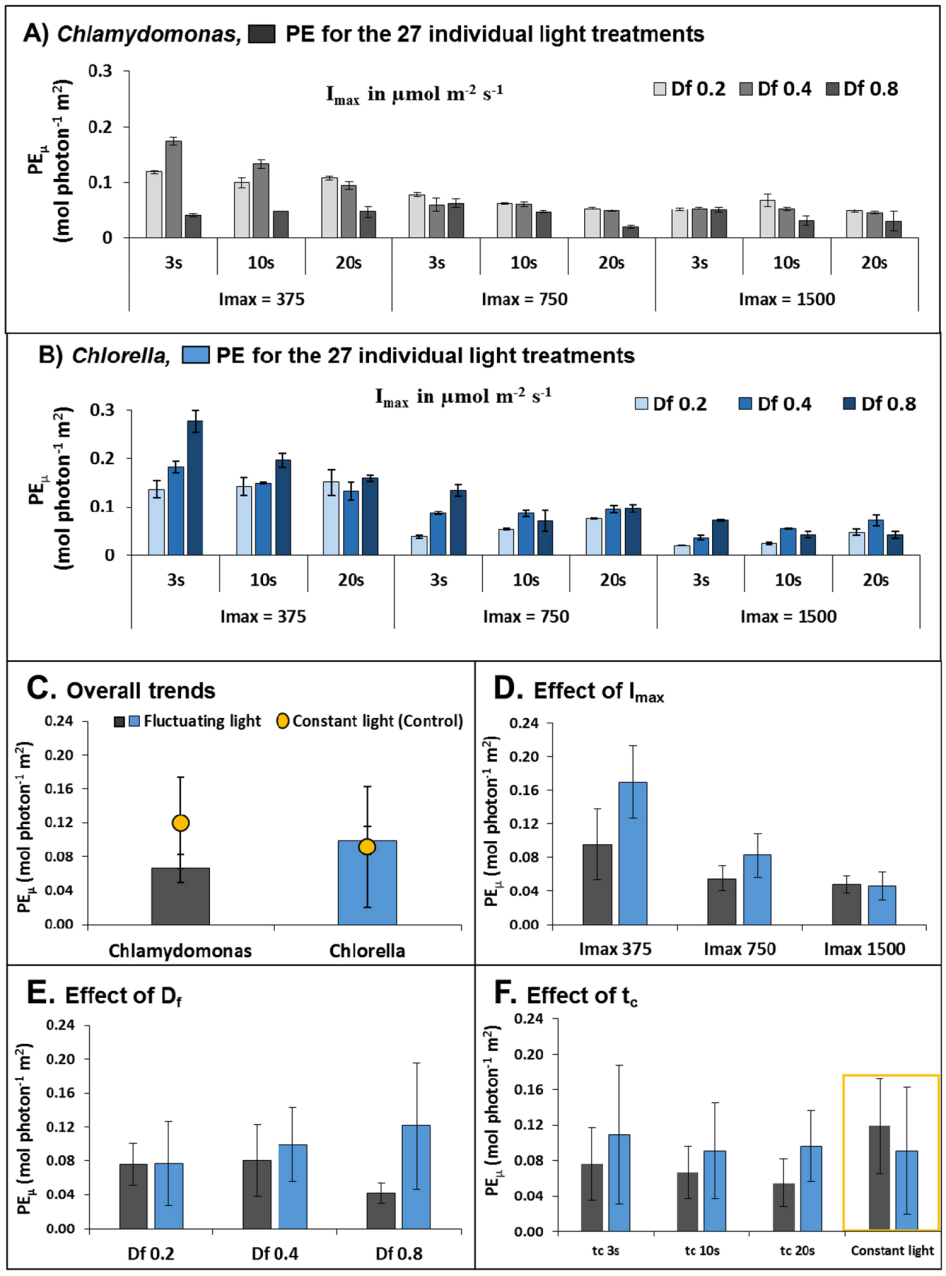
Trends in photosynthetic efficiency (PE_µ_) under different light regimes of *Chlamydomonas* (grey bars) and *Chlorella* (blue bars). (**A** and **B**) individual PE_µ_ data of the 27 light treatments for *Chlamydomonas* and *Chlorella*, respectively (*n* = 3), (**C**) the overall trends in averaged PE_µ_ values over all conditions of D_f_, I_max_ and t_c_ tested (*n* = 27), (**D**) the averaged PE_µ_ values of D_f_ and t_c_ combined to show effect of I_max_ (*n* = 9), (**E**) the averaged PE_µ_ values of I_max_ and t_c_ combined to show effect of D_f_ (*n* = 9) and (**F**) the averaged PE_µ_ values of D_f_ and I_max_ combined to show effect of t_c_ (*n* = 9). Error bars represent the standard deviation (SD) of individual treatments within biological triplicates (A,B) and between different treatments (**C–F**).

A further dataset with a D_f_ of 0.6 (at each level of I_max_ and t_c_) provided 9 independent data points used for model validation and goodness of fit (Table 1, validation data are indicated by ‘*’. See section (Model validation shows that the light factors D_f_, I_max_ and t_c_ can be used to predict PE_μ_ accurately in *Chlorella* and moderately in *Chlamydomonas*) for results). For all treatments, the combination of each D_f_ and I_max_ also resulted in 12 unique integrated average irradiance levels, I_avg_ (mol photons m^−2^ h^−1^). Additional experiments compared the PE_µ_ between cells exposed to fluctuating regimes with cells exposed to constant illumination (control) with the same I_avg_ to compare the effect of light regime and light dose (Fig. 2C, Supplementary Table S1, Supplementary Fig. S4).

Light screen experiments were conducted over 3 days in a controlled semi-continuous cultivation regime. As light acclimation occurs on a timescale of several hours to days, sufficient time was given for the cells to acclimate to the light regime that they were exposed to. To minimise cell shading effects with increasing OD, cultures were diluted back to the same initial OD_750_ of 0.1 (pathlength 5 mm) each day. Quasi-steady-state growth rates, μ (h^−1^) were calculated (Equation 3) from 3-hourly OD_750_ measurements (Supplementary Figs S1 and S2) on Day 2 during the exponential phase (after ~38 hours of light regime exposure) and normalised to the light received to estimate the photosynthetic efficiency (PE_μ_) (Equation 4).

### Photosynthetic efficiency under different light regimes

The PE_µ_ of *Chlamydomonas* and *Chlorella* under all 27 fluctuating light regimes are shown in Fig. 2A and B. Some similarities in the general trends of *Chlamydomonas* and *Chlorella* are evident, such as the effect of I_max_, where a large increase in PE_µ_ occurred with decreasing I_max_. To better depict PE_µ_ trends, individual treatments were averaged for each species over all factors (Fig. 2C), and over all but one factor (Fig. 2D–F). Overall, *Chlorella* exhibited a ~50% higher PE_µ_ than *Chlamydomonas* (average PE_µ_ of 0.099 ± 0.060 mol photon^−1^ m^2^ and 0.066 ± 0.034 mol photon^−1^ m^2^ respectively, Fig. 2C), in line with previous reports^32^.

Figure 2C also shows the mean PE_µ_ obtained under constant light was ~80% higher in *Chlamydomonas* but approximately the same for *Chlorella* (−7.5%) than that obtained under fluctuating light of the same I_avg_. For *Chlamydomonas*, this result concurs with other studies showing a negative impact of fluctuating light on time-integrated photosynthesis and growth rates^10,12,33,34^. Interestingly, for this strain of *Chlorella,* fluctuating light had little effect compared to constant light conditions.

For main effects of each factor, Fig. 2D shows at the lowest I_max_ value, the mean PE_µ_ increased up to two-fold for *Chlamydomonas* and 3.67-fold for *Chlorella*, respectively, indicating that photosynthetic light utilisation is compromised under high incident light (i.e. at noon under outdoor conditions)^35–37^, especially for *Chlorella*.

The trends of D_f_ (Fig. 2E) resulted in diametrically opposing responses: PE_µ_ in *Chlamydomonas* performed best at a low D_f_ (increasing up to 83% from D_f_ = 0.8 to D_f_ = 0.2) while *Chlorella* at a high D_f_ (PE_µ_ increased up to 58% from D_f_ = 0.2 to D_f_ = 0.8). Since mass cultures operating under high cell densities is advantageous to reduce downstream processing costs, these results suggest that *Chlorella* is more suited to mass cultivation than *Chlamydomonas*.

For both species, the effect of t_c_ seemed minor (Fig. 2F). Cell cycling in the range analysed (t_c_ = 3, 10, 20 s) exhibited a modest increase in PE_µ_ with decreasing t_c_ values (39% for *Chlamydomonas* and 13% for *Chlorella*). While large improvements of PE_µ_ have been reported under sub-second cycle times approaching the ‘flashing light effect’^28,38,39^, this is in line with other studies that have reported similar modest improvements for *Chlamydomonas* below cycle times of 10 s^12^ and little effect in the seconds range for other *Chlorella* sp. and other algae^11,38^.

### Modelling light factor interactions using response surface methodology

Response surface methodology of the complete factorial design^40–45^ was next employed to model and explore the interactions between the three input factors (I_max_, D_f_ and t_c_) to PE_µ_. Furthermore, to determine the influence of photoregulation under fluctuating light on PE_µ_, supporting parameters at the level of PSII regulation for *Chlamydomonas* and *Chlorella* were also modelled from chlorophyll fluorescence data. These are: the operating efficiency of PSII (ϕPSII) – a measure of the proportion of absorbed light used for photochemistry; maximum quantum efficiency of PSII photochemistry (F_v_/F_m_) – an indicator of PSII inactivation via photoinhibition; and non-photochemical quenching (NPQ) – the apparent rate constant for heat loss from PSII^44^. These parameters provide clues as to the underlying mechanisms of the observed PE_μ_.

The three levels of each factor (Table 1) were coded with the mid-point (coded as ‘0’) and this was halved and doubled in the experimental design such that the coded factors of the independent variables were calculated using the logarithmic equation,

1 $${{x}}_{{\boldsymbol{i}}}=(1.4427\,\mathrm{ln}({{X}}_{{\boldsymbol{i}}})+{{A}}_{{\boldsymbol{i}}})$$

where, *x* is the coded factor level, *X* is the actual value of the factor, i = 1, 2, 3*;* A is the intercept value of the logarithmic function for each factor with A_1_ = 1.3219, A_2_ = −9.5507 and A_3_ = −3.3219 for D_f_, I_max_ and t_c_ respectively.

Quadratic models (Equation 2) were fitted to the data:

2 $$Y={\beta }_{0}+\,\sum _{i=1}^{k}{\beta }_{i}{x}_{i}+\sum _{i=1}^{k-1}\sum _{j=i+1}^{k}{\beta }_{ij}{x}_{i}{x}_{j}+\,\sum _{i=1}^{k}{\beta }_{ii}{x}_{i}^{2}$$

In Equation 2, Y is the predicted response variable (PE_µ_, ϕ_PSII_, F_v_/F_m_ or NPQ); β_0_, β_i_, β_ij_ and β_ii_ are the coefficients for intercept, linear, interaction and quadratic effects respectively; *x*_1_*, x*_2_ *… x*_*k*_ are the coded values of the input factors (i ≠ j); and k = 3. Multiple regression of the data was used to obtain the regression coefficients.

#### Model validation shows that the light factors I_max_, D_f_ and t_c_ can be used to predict PE_µ_ accurately in *Chlorella* and moderately in *Chlamydomonas*

For the primary response, PE_µ_, the quadratic model demonstrated a moderate and high goodness of fit for *Chlamydomonas* (R^2^ = 0.67) and *Chlorella* (R^2^ = 0.93), respectively.

To assess whether the model fit was adequate to predict PE_µ_ within the range analysed, the quadratic models were validated using an additional set of experimental data at D_f_ = 0.6 at each level of I_max_ and t_c_ (9 experimental sets for each strain) (Table 1). Comparing the fitted models against the actual data gave a low R^2^ of 0.456 for *Chlamydomonas* and a high R^2^ of 0.882 for *Chlorella* (Supplementary Fig. S5). In general, the residuals showed a normal distribution and the Cook’s distance plot showed only a small number of outliers for *Chlamydomonas* and *Chlorella* (Supplementary Fig. S5).

For Chlorella, these results indicated that the three light factors accounted for a high proportion of variation in PE_µ_ observed and can be used to adequately predict their relationship to PE_µ_. For *Chlamydomonas,* it seems there are more complex regulations of the photosynthetic machinery, which cannot be modelled with these factors alone.

#### The light factors of I_max_ and D_f_ significantly affect PE_µ_ under fluctuating light

**Figure 3:**
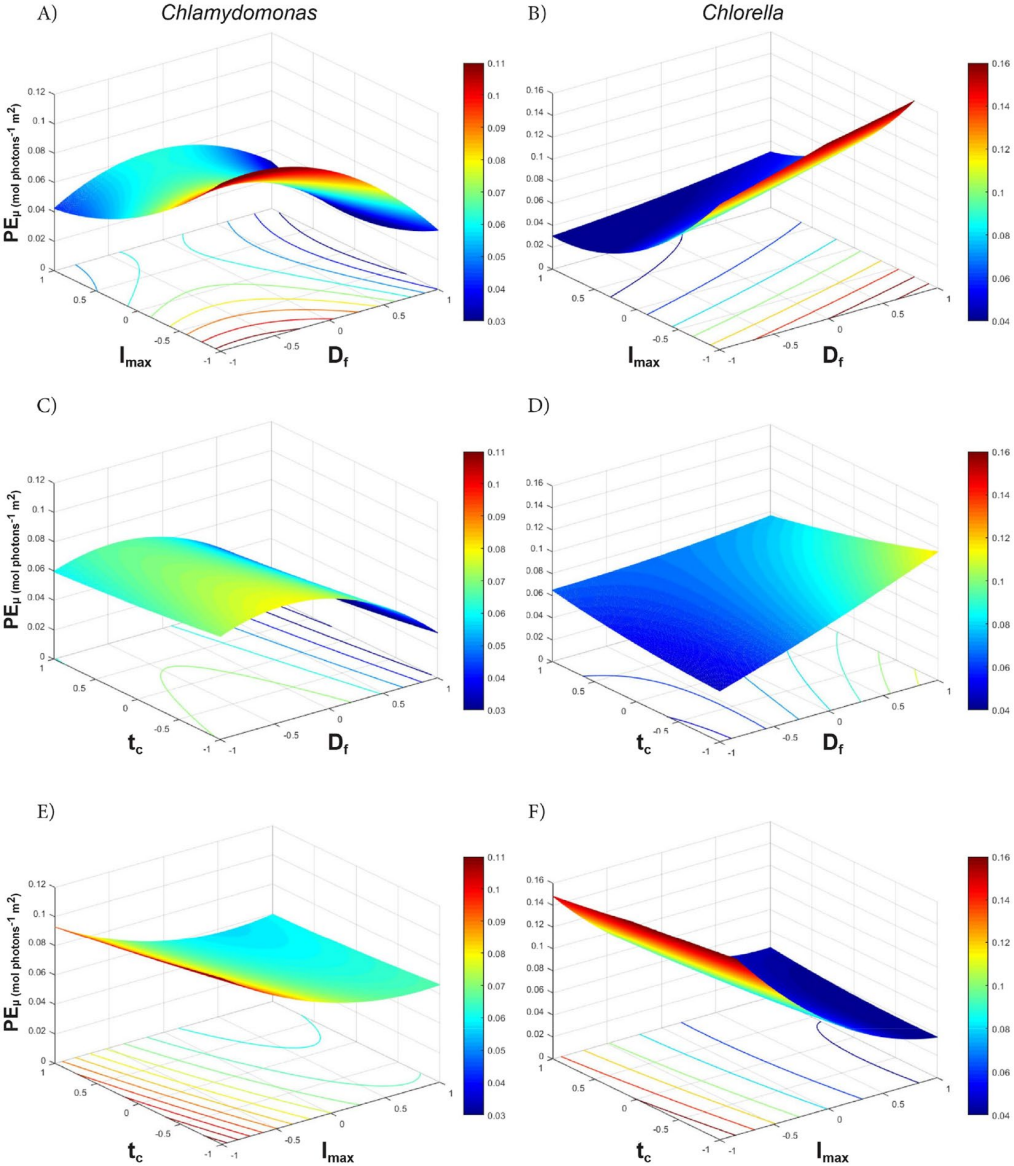
Response surface (3D) and contour (2D) plots of two-way interactions of factors affecting the PE_µ_ (mol photon^−1^ m^2^) of *Chlamydomonas* (**A,C,E**) and *Chlorella* (**B,D,F**). The colour bar depicts high PE_µ_ values in red and lower PE_µ_ values in blue.

The coefficient terms tabulated in Table 2 show the relative size and direction that effect each factor has on the response variables, while the three dimensional (3D) response surface plots and 2D contour plots graphically depict the interactions of two factors on the primary response of PE_µ_, where the third factor is set to the midpoint (Fig. 3).

For *Chlamydomonas*, the most significant factors affecting PE_µ_ were: I_max_ (p-value = 3.83E^−08^), D_f_ (p-value = 1.04E^−08^), and the interaction of D_f_-I_max_ (p-value 1.05E^−04^) (Table 2). Here, both high D_f_ and high I_max_ had similar negative impacts on PE_µ_, yet the interaction of D_f_-I_max_ had a positive effect, suggesting that dense cultures may offer some protection under high light whilst dilute cultures may improve PE_µ_ under low light. As expected, the 3D plots show the highest PE_µ_ values at a combination of low D_f_ (i.e. not light limited) and low I_max_ (i.e. not photo-inhibited) (Fig. 3A), however, the slight saddle shape of the interaction plot at high I_max_ shows that the optimal D_f_ is around 0.4 (at the mid-point) for *Chlamydomonas*.

The PE_µ_ of *Chlorella* was most significantly adversely affected by high I_max_ (p-value 9.92E^−37^), and unlike *Chlamydomonas*, showed a significant positive response for increasing D_f_ (p-value 4.67E^−12^). The I_max_-D_f_ interaction showed an exponential increase in PE_µ_ with a reduction of I_max_ and an increase in D_f_ (Fig. 3B). However, the significant negative interaction of D_f_-t_c_ (Table 2) suggests that long cycle times could adversely affect productivity in high density cultures (Fig. 3D). Overall, for *Chlamydomonas* a low I_max_ and low D_f_ (Fig. 3A) and for *Chlorella* a low I_max_ and high D_f_ (with moderate benefits of low t_c_) (Fig. 3 B and D) resulted in the highest PE_µ_.

### PSII regulation has a strong effect on PE_µ_ under fluctuating light

To assess some underlying mechanisms that may affect PE_µ_, chlorophyll fluorescence measurements were taken to assess levels of stress and photo-inhibition (F_v_/F_m_), the operating efficiency of PSII (Φ_PSII_) and non-photochemical quenching (NPQ). The data was fitted to the quadratic model (Equation 2) to compare the magnitude of effect of the three light factors. Additionally, changes in the ratio of OD_680_/OD_750_ were used as a high-throughput proxy to determine photoacclimation via changes in chlorophyll content.

A high goodness of fit to the quadratic model was observed in *Chlamydomonas* for Φ_PSII_ (R^2^ = 0.89) and F_v_/F_m_ (R^2^ = 0.74) and, in *Chlorella*, for F_v_/F_m_ (R^2^ = 0.91), suggesting that PSII regulation is highly affected by the three light factors examined in this study and is a contributing factor to the observed PE_µ._ Remarkably, all treatments for both species showed low NPQ (<0.3) relative to average values reported in literature (up to ~2 for *Chlamydomonas* and ~1.5 for Chlorella)^15,46–48^ and a poor goodness of fit to the quadratic model for both strains (see Supplementary Table S2). Other stressors, such as nutrient limitation, are also known to increase NPQ^49^. Since both strains were cultivated on previously optimised nutrients this may have contributed to the overall reduced NPQ in this study.

**Figure 4:**
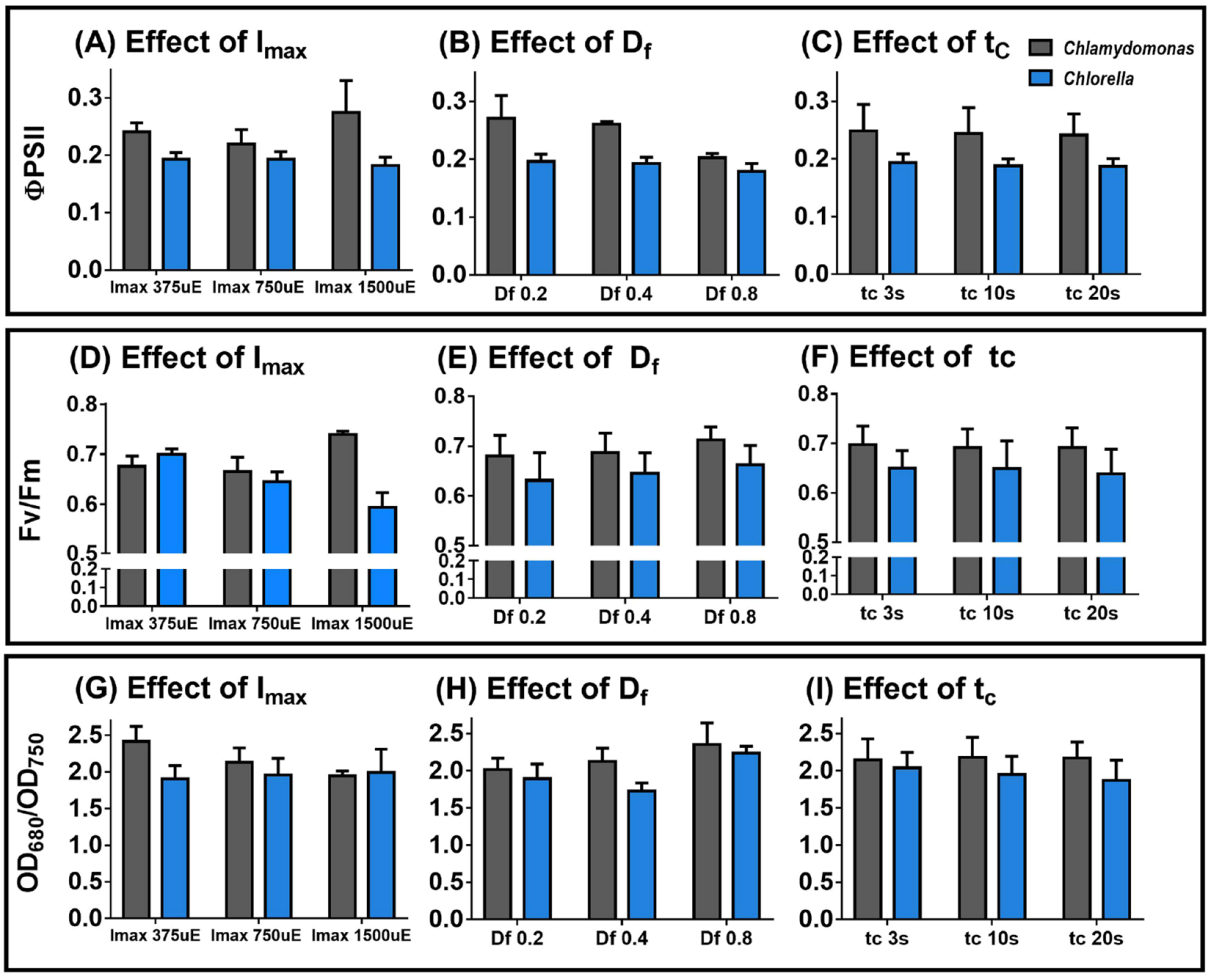
Trends in underlying photosynthetic mechanisms. Plots depict averaged effects of I_max_, D_f_ and t_c_ on ΦPSII (**A,B** and **C**) (n = 2); F_v_/F_m_ (**D,E** and **F**) and OD_680_/OD_750_ (**G,H** and **I**) respectively for *Chlamydomonas* (grey bars) and *Chlorella* (blue bars) (n = 3, Error bars represent standard deviation).

For *Chlamydomonas*, a significant (p-value = 1.79E-17) reduction in Φ_PSII_ occurred at high D_f_ (Table 2, Fig. 2E). This suggests that efficient electron transfer is compromised under high dark fractions for this alga and links Φ_PSII_ to the reduced PE_µ_ trends under high D_f_ observed. Furthermore, increased OD_680/750_ measurement (a proxy for chlorophyll content per cell) was prominent with increasing D_f_ (Fig. 4H), suggesting high dark fractions lead to increased cellular chlorophyll levels typical for low-light acclimation, which may further explain the lower efficiency of light utilisation (i.e. PE) at high D_f_ (Fig. 2E). Remarkably, a high I_max_ actually improved both Φ_PSII_ (Fig. 4A) and F_v_/F_m_ (Fig. 4D) and lowered OD_680/750_ (Fig. 4G), despite a reduction in PE_µ_ (Fig. 2D). This suggests that while photosynthetic rates improved in *Chlamydomonas* under high light, the over-saturating irradiance could not be fully utilised by the Calvin-Benson cycle, suggesting other downstream mechanisms such as alternative electron sinks^50^ could become relevant under high light.

For *Chlorella*, the most significant factor corresponding directly to PE_µ_ was the effect of I_max_ on F_v_/F_m_, which gave a large negative coefficient in the model (Table 2) and showed a noticeable decline in F_v_/F_m_ with increasing I_max_ (Fig. 4D). Like *Chlamydomonas*, increasing D_f_ was found to have a positive effect on F_v_/F_m_ (Fig. 4E), also seen by the relative magnitudes of coefficients and their significance (p-value = 3.09E-07), and a significant positive interaction between D_f_ − I_max_ (p-value = 5.19E-03)_._ Similar to *Chlamydomonas, Chlorella* exhibited an up-regulation of OD_680/750_ (indicative of higher chlorophyll) at high D_f_ (Fig. 4H, Supplementary Table S2).

In summary, these results suggest that *Chlorella* is sensitive to high light as seen by PSII inactivation but less sensitive to light/dark fluctuations. In contrast, *Chlamydomonas* is sensitive to strong light/dark fluctuations due to disrupted electron transport flows but seems to have better acclimisation strategies to cope with high light. These results suggest that maintaining *Chlamydomonas* at relatively dilute cultures is beneficial, whereas operating *Chlorella* at high densities is preferable, especially under high light.

### Optimisation predicts a two-fold higher maximum PE_µ_ for *Chlorella* compared to *Chlamydomonas*

It is evident from the 3D surface plots (Fig. 3) showing PE_µ_ response that the maxima occur at the extremes in most instances. The maximum PE_µ_ values (at the mid-point, i.e. level 0) and their corresponding factor levels were used to obtain the maximum PE_µ_ and optimum conditions. For both *Chlamydomonas* and *Chlorella*, the maximum PE_µ_ values occurred at the minimum I_max_ (375 µE) and the minimum value of t_c_ (Table 3). Using this combination of I_max_ and t_c_, the optimal D_f_ values were found to be 0.24 and 0.8 for *Chlamydomonas* and *Chlorella* respectively. These combination of factor values results in a theoretical maximum PE_µ_ of 0.126 and 0.226 mol photon^−1^ m^2^ (Table 3), predicting a nearly 2-fold higher maximum PE_µ_ for *Chlorella* than *Chlamydomonas*. As discussed in the section 3.3.1, the three light factors modelled only explains two thirds of the variation in PE_µ_ for *Chlamydomonas* and these results are indicative only for this species.

**Table 3:** Optimisation of PE_µ_ and the respective factor levels around the mid-point of each factor, and around the optimised point for total predicted maximum PE_µ_ within the ranges of the full factorial design.

Species	Condition	Predicted max PE_µ_	D_f_		I_max_		t_c_	
		(mol photon^−1^m^2^)	Coded	(−)	Coded	(µmol m^−2^ s^−1^)	Coded	(s)
*Chlamydomonas*	t_c_ midpoint	0.116	−0.75	0.24	−1	375	0	10
	I_max_ midpoint	0.079	−0.4	0.30	0	750	−1	5
	D_f_ midpoint	0.113	0	0.40	−1	375	−1	5
	Optima	0.126	−0.73	0.24	−1	375	−1	5
*Chlorella*	t_c_ midpoint	0.194	1	0.80	−1	375	0	10
	I_max_ midpoint	0.117	1	0.80	0	750	−1	5
	D_f_ midpoint	0.178	0	0.40	−1	375	−1	5
	Optima	0.226	1	0.80	−1	375	−1	5

## Concluding Remarks

The HTS coupled with response surface methodology delivers a working statistical design for simultaneous light optimisation of several species of microalgae. This platform has been used to screen growth responses to nutrients and organic carbon sources^20,23^, and can be extended to screen other parameters such as CO_2_ or growth contaminants (e.g. herbicides, antibiotics, bacteria or predating organisms), and could monitor other response variables such as lipid accumulation (e.g. Nile Red) and protein expression using fluorescence tags. Some limitations imposed by the microwell HTS can include high variation between replicates when trialled at conditions that give very low growth rates; and some evaporation losses that limit the duration of the experiment due to the low culture volume. Radzun, K. A. *et al*. have reported that despite some evaporative losses observed in the TECAN robotic system, the RSD values were considerably lower than can be achieved through manual measurement.

As the OD measurements in the plate reader are made vertically rather than horizontally, the reduction of depth due to evaporation is compensated for by the concomitant increase in cell concentration to maintain the same optical pathlength^23^. Furthermore, variation can be reduced by adding additional technical replicates (as done in this study), while evaporation can be addressed by using a humidifier in the enclosed chamber system (currently being developed) and/or reducing the frequency of measurement readings which requires lid removal. Despite this, the HTS provides a cost-effective, rapid and efficient platform to obtain large data-sets for a wide array of solar driven microalgae applications, which would otherwise require significant investment of time, money and resources.

In most mass cultures, particularly those of outdoor raceway ponds, severe light limitation exists, typically where light penetrates only the first millimetres or centimetres at most and high dark fractions of 90% or greater are normal^24,30^. These dark fractions and cycling between light/dark zones can be detrimental for redox imbalances, as was shown to be the case for *Chlamydomonas*. Therefore, species such as the strain of *Chlorella* tested here, have a selective advantage for mass culture, as productivity was found to be unaffected by light fluctuations. Furthermore, it opens up new insights for the design of high efficiency cell lines, capable of handling both high light intensities and strong light/dark fluctuations. Improving light distribution deeper within the culture depth with minimal transmittance losses (e.g. by increasing surface to volume ratios or using specially designed light guides^51^) may be another strategy to improve PE_µ_, rather than adjusting cycle time (by increasing mixing rates, gas sparging) particularly as the latter would require higher energy inputs with minimal gains in PE_µ_. Another important deduction of strain-specific characterisation for scale up was the detrimental effect of cycle time on PE_µ_ for *Chlamydomonas* (~−46%) versus a similar effect for *Chlorella* as compared to constant light. This signifies the application of our HTS outcomes toward strain selection as well as growth platform selection (i.e. open pond (slow mixing) *versus* tubular PBRs (faster mixing) or other designs) when going from laboratory (constant light) to outdoor systems (fluctuating light). In both alga, as is typical of other species, high incident light has the most detrimental effect on PE_µ_. Therefore, efforts to diffuse light sources, such as done through the use of reflectors, or to use vertical flat panels or vertically stacked tubular photobioreactors to avoid direct sunlight at high light periods, may benefit from the ‘light dilution effect’.

Previous transcriptomic and proteomic studies in *Chlamydomonas* have shown that acclimation to environmental stimuli is achieved by remodelling photosystem I and II antenna complexes, further highlighting the flexibility of their photosynthetic machinery^52^. While *Chlamydomonas* may possess the survival strategies required to acclimate to changing light conditions, typically for soil environments, they may not be tuned for high biomass productivity, unlike fast-growing strains like the *Chlorella* strain used in this study, which despite seemingly lacking the level of regulatory sophistication, might be better suited for mass cultivation.

In conclusion, the HTS method developed here enables a rapid approach to optimise systems design, scale up operational conditions and species selection to advance feasible solar-driven biotechnologies.

## Materials and Methods

### Strains and pre-culture conditions

Liquid pre-cultures were prepared in triplicate (40 mL culture in 100 ml flasks) and inoculated with either *C. reinhardtii* WT strain CC125^53^ or *Chlorella* sp. 11_H5^19^ (Australian isolate) maintained on TAP^54^ agar (1.5%) plates. To ensure nutrients were non-limiting, photoautotrophic medium previously optimised for each species was used for *C. reinhardtii* (PCM^55^, N source NH4^+^) and *Chlorella* sp (OpM_2_^20^, N source urea). Flasks were maintained on shakers (200 rpm) in an enclosed incubation system at 23 °C, 1% CO_2_ and a 16/8 hour light/dark cycle, illuminated with 100 µmol m^−2^ s^−1^ of overhead white fluorescent light for 5 days.

To ensure that the cultures were well synchronised to the light conditions being tested, flask pre-cultures first acclimated to a 16/8 h light/dark cycle were inoculated into microwell plates (150 µL), and gradually acclimated to the light intensity close to the mean I_avg_ before the first measurement. For the higher light intensity experiments (I_max_ = 1500 µmol m^−2^ s^−1^), care was taken not to shock the low density cultures by subjecting them to a step-wise gradually increasing light regime (a detailed summary of the acclimation regimes is provided in Supplementary Table S3).

### Automated HTS and lighting design

The design, structure and operation of the HTS system (Tecan Freedom Evo 150, Tecan Group Ltd., Männedorf, Switzerland) is as previously described^20,23^. Briefly, the HTS system is an enclosed chamber fitted with three orbital shakers which hold six microwell plates each, a robotic manipulator arm that removes the plate lid and carries the plates to a reader (Infinite M200 PRO, Tecan Group Ltd., Männedorf, Switzerland, Fig. 1C) and atmospheric CO_2_ control. Each of the 18 microwell plate positions is fitted with 96 ‘warm white’ LEDs positioned directly under each well of a 96-well plate. Each of the LED arrays is controlled by user defined scripts on an Adruino® integrated circuit controller and software, permitting 18 different light conditions to be tested in parallel. LEDs were fitted with a low pass LC filter to smooth the intensity signal from pulse width modulation to variable voltage, thereby eliminating ‘flashing light’ phenomena due to on/off signals. The spectrum of wavelengths of LEDs is compared against that of natural sunlight (see Supplementary Fig. S6). For simplicity, a sinusoidal mixing regime was assumed to allow tight control of the factors of I_max_, D_f_ and t_c_, as has been used in previous studies^56,57^. Pre-cultures were centrifuged (500 *g*, 20 min, 18 °C) and the pellet re-suspended in fresh medium. To minimise cell shading effects and ensure tight light control, a volume of 150 µl was chosen for a short pathlength of 5 mm and a semi-continuous cultivation regime was applied by daily culture dilutions back to a starting OD_750_ of 0.1. Each of the three biological replicates per species was inoculated into each well of a 96-well plate. Since only two strains were tested in this study, all wells were inoculated, providing 14 technical replicates per biological replicate. Of these, 10 wells were used for automated OD_750_ and OD_680_ readings, the remaining wells (of two biological replicates) were extracted on day 2 for manual PSII measurements. The final row of 12 wells contained 150 µl pure media to use as blank controls.

### Growth rate and photosynthetic efficiency (PE_µ_) measurements

Growth rates were calculated from 3-hourly OD_750_ measurements. High-throughput automated measurements of OD_750_ were used as a proxy for growth from which growth rates, *µ* (h^−1^), were calculated as the rate of change of OD_750_,

3 $$\mu =({ln}\,O{D}_{750}({t}_{2})-\,{ln}\,O{D}_{750}({t}_{1}))/({t}_{2}-\,{t}_{1})$$

where, t_1_ and t_2_ are the time points at which OD_750(t1)_ and OD_750(t2)_ were measured.

A 3-hour measuring frequency during the light period was used for the growth curve calculations. This frequency was chosen to limit evaporation and contamination issues. A detailed description of the growth curves, sampling points and lighting schedule can be found as Supplementary Figs S1 and S2.

The main response variable, PE_µ_, was assumed to be indicative of light utilisation efficiency of the microalgae, where the growth rate normalised to the average integrated PAR received,

4 $$PE=\mu /{I}_{avg}$$

And the I_avg_ is,

5 $${I}_{avg}=\,{\int }_{0}^{t}I(t)dt\ast 3.6\ast {10}^{-9}$$

In Equation 5, t_c_ is the cycle time, *I*(t) is the irradiance (µmol photons m^−2^ s^−1^) at a given time of t_c_, and 3.6*10^−9^ is the conversion factor from µmol photons m^−2^ s^−1^ to mol photons m^−2^ h^−1^.

### Chlorophyll fluorescence of photosystem II measurements

Photosystem II (PSII) kinetics were measured as a function of PSII chlorophyll fluorescence^10,58,59^. Biological duplicates of each sample (dilution factor of 5) was added to a Fluorimeter cuvette (Sigma), dark adapted for 20 minutes and processed using the FluoroWin software (Photon Systems Instruments, Czech Republic). The quenching analysis protocol had the following settings: measuring light: 20% V; saturating pulse: 0.9 s, 80% V; actinic light: 51 s, 18.3 V (~800 µmol m^−2^ s^−1^). Weak infrared pulses (730 nm) were applied for 5 s prior to measurement to quench Q_A_. The PSII parameters calculated from the quenching analysis were: *F*_*v*_*/F*_*m*_ (maximum quantum efficiency of PSII), *Φ*_*PSII*_ (PSII operating efficiency), and *NPQ* (Non photochemical Quenching) using respectively,

6 $${F}_{v}/{F}_{m}=({F}_{m}-\,{F}_{0})/{F}_{m}$$

7 $${\phi }_{PSII}=({F}_{m}^{\text{'}}-F)/{F}_{m}^{\text{'}}$$

8 $$NPQ=({F}_{m}/{F}_{m}^{\text{'}})-\,1$$

### Photoacclimation via OD_680/750_

Chlorophyll *a* has a maximum absorbance at 680 nm. Therefore, OD_680_ measurements were normalised to OD_750_ (OD_680/750_) as a proxy of changes in chlorophyll absorption between different light regimes.

### Statistical Analysis

All data are expressed as Mean ± SD of three biological replicates (for automated readings) and two biological replicates (for the manual PSII measurements), each with multiple technical replicates as mentioned in section 5.2. MATLAB was used for the design and analysis of the response surface methodology. A p-value <0.05 was used for determining significant effects. Both contour and surface plots were developed for visualisation of the data and to predict the relationship and interaction effects on the light utilisation efficiency. Regression coefficient (R^2^) was used to resolve the goodness of fit. The fitted model using the regression coefficients was validated with an additional experimental dataset.

## Electronic supplementary material

Supplementary information for Manuscript
