# Supplementary material for: High-throughput optimisation of light-driven microalgae biotechnologies
Source: Sci Rep. 2018 Aug 3;8:11687. doi: 10.1038/s41598-018-29954-x (PMC6076246; doi:10.1038/s41598-018-29954-x)
Supplement: Supplementary file 1 — Supplementary information for Manuscript [file 41598_2018_29954_MOESM1_ESM.pdf]

Supplementary information for Manuscript

Title: High-throughput optimisation of light-driven microalgae biotechnologies

Shwetha Sivakaminathan[1], Ben Hankamer[1], Juliane Wolf[1*], Jennifer Yarnold[1*]

[1]The University of Queensland, Institute for Molecular Bioscience, 306 Carmody Road, St Lucia, Australia

Contents

Supplementary Figure S1 for Section 3.2 (a) 1
Supplementary Figure S2 for Section 3.2 (b) 2
Supplementary Figure S3 for Section 3.2 (c) 3
Supplementary Table S1 for Section 3.2 4
Supplementary Figure S4 for Section 3.2 (d) 5
Supplementary Figure S5 for Section 3.3.1 6
Supplementary Table S2 for Section 3.4 7
Supplementary Table S3 for Section 5.1 8
Supplementary Figure S6 for Section 5.2 9

Supplementary Figure S1 for Section 3.2 (a)

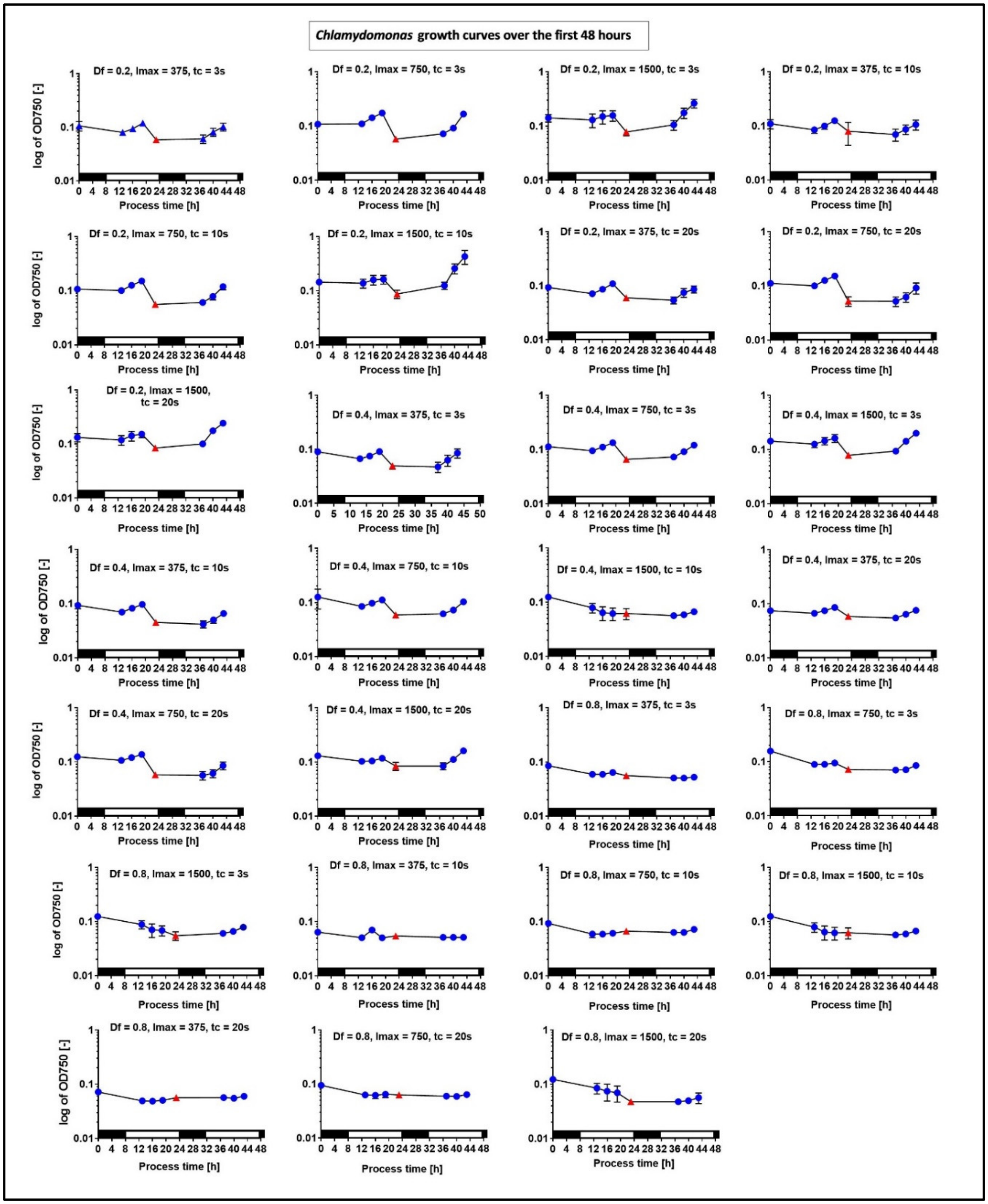

Supplementary Figure S1. Growth curves of *Chlamydomonas* over the first 48 hours for the 27 conditions tested. Red triangles represent the OD measurement after dilution. Bottom bars represent the duration of the 16/8 h light/dark cycles: black = dark period and white = light period with the respective fluctuating light regime. The points above the first white bar represent Day 1 (acclimation phase) and the points after the red triangle represent Day 2 (quasi-steady state) data used for growth rate and PE_{μ} calculations. All data are mean of 3 replicates ± standard deviation.

Supplementary Figure S2 for Section 3.2 (b)

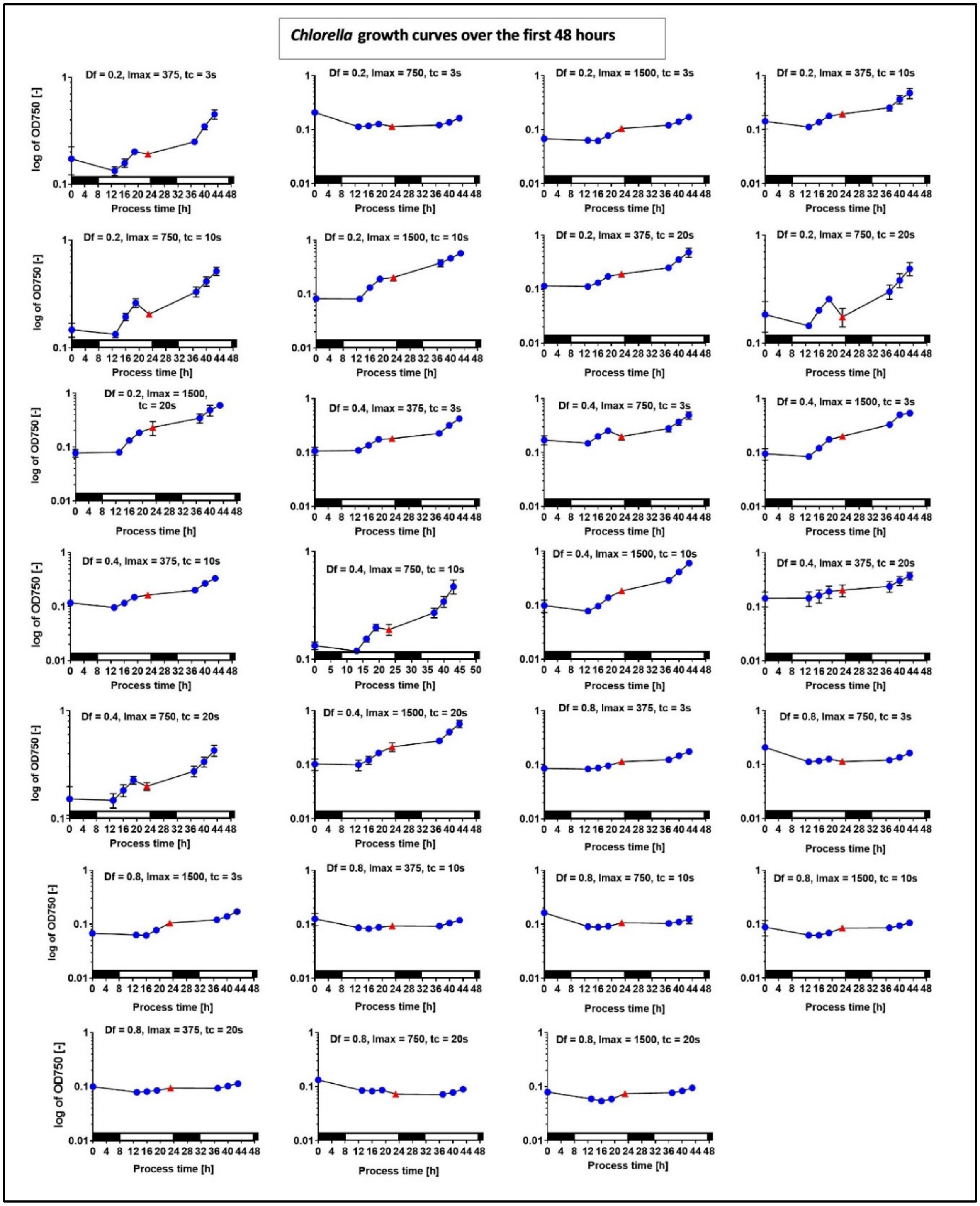

Supplementary Figure S2. Growth curves of *Chlorella* over the first 48 hours for the 27 conditions tested. Red triangles represent the OD measurement after dilution. Bottom bars represent the duration of the 16/8 h light/dark cycles: black = dark period and white = light period with the respective fluctuating light regime. The points above the first white bar represent Day 1 (acclimation phase) and the points after the red triangle represent Day 2 (quasi-steady state) data used for growth rate and PE_{μ} calculations. All data are mean of 3 replicates ± standard deviation.

Supplementary Figure S3. Trends in photosynthetic efficiency (PE_{μ}, mol photon^{-1} m^{2}) under the 27 different light regimes for Chlamydomonas (grey bars) and Chlorella (blue bars) during the acclimation phase (Day1, A and B).

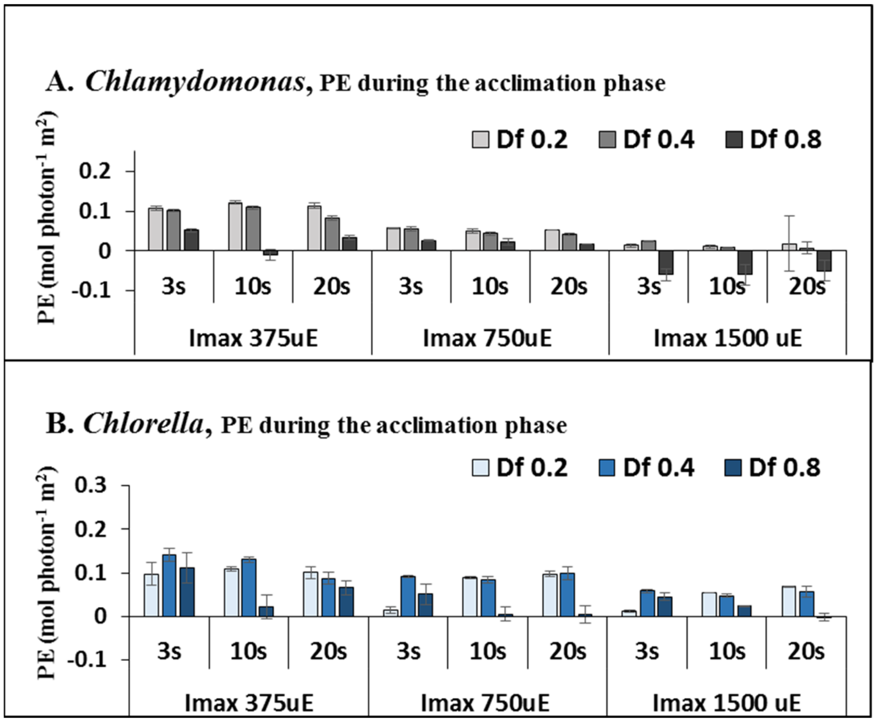

Supplementary Table S1 for Section 3.2

Supplementary Table S1. PE_μ of *Chlamydomonas* and *Chlorella* under constant light of the same average irradiance as fluctuating light treatments of each unique combination of I_{max} and D_f (as per Table 1 in the main text). All data represent the mean of 3 replicates ± standard deviation

I_{avg}	PE_μ under constant light (mol photon^{-1} m^2)	
(mol m^{-2} h^{-1})	*Chlamydomonas*	*Chlorella*
0.619	0.149 ± 0.004	0.120 ± 0.019
0.490	0.169 ± 0.008	0.151 ± 0.006
0.367	0.175 ± 0.008	0.178 ± 0.010
0.180	0.178 ± 0.040	0.253 ± 0.026
1.242	0.054 ± 0.004	0.103 ± 0.003
0.979	0.113 ± 0.000	0.058 ± 0.007
0.738	0.116 ± 0.013	0.078 ± 0.002
0.360	0.197 ± 0.006	0.050 ± 0.002
2.480	0.045 ± 0.010	0.011 ± 0.000
1.958	0.061 ± 0.004	0.029 ± 0.001
1.472	0.055 ± 0.004	0.051 ± 0.001
0.713	0.111 ± 0.010	0.049 ± 0.005

Supplementary Figure S4 for Section 3.2 (d)

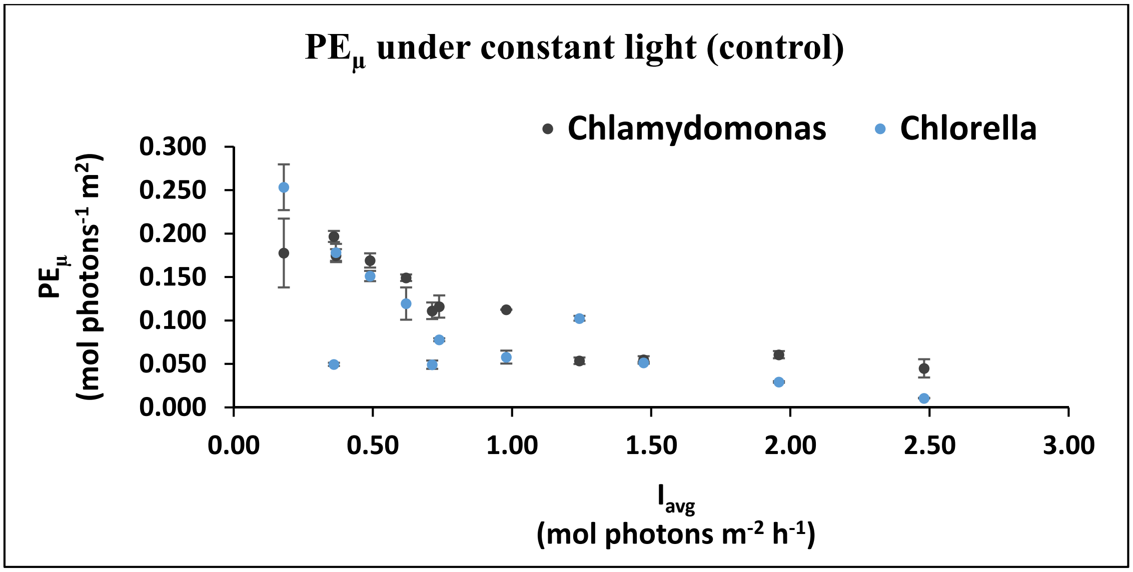

Supplementary Figure S4. Trends in photosynthetic efficiency (PE_μ, mol photon^{-1} m^2) under constant light for *Chlamydomonas* and *Chlorella* of the same I_{avg} as fluctuating light regimes of each unique combinations of I_{max} and D_f on Day 2. Error bars represent standard deviations (n=3).

Supplementary Figure S5 for Section 3.3.1

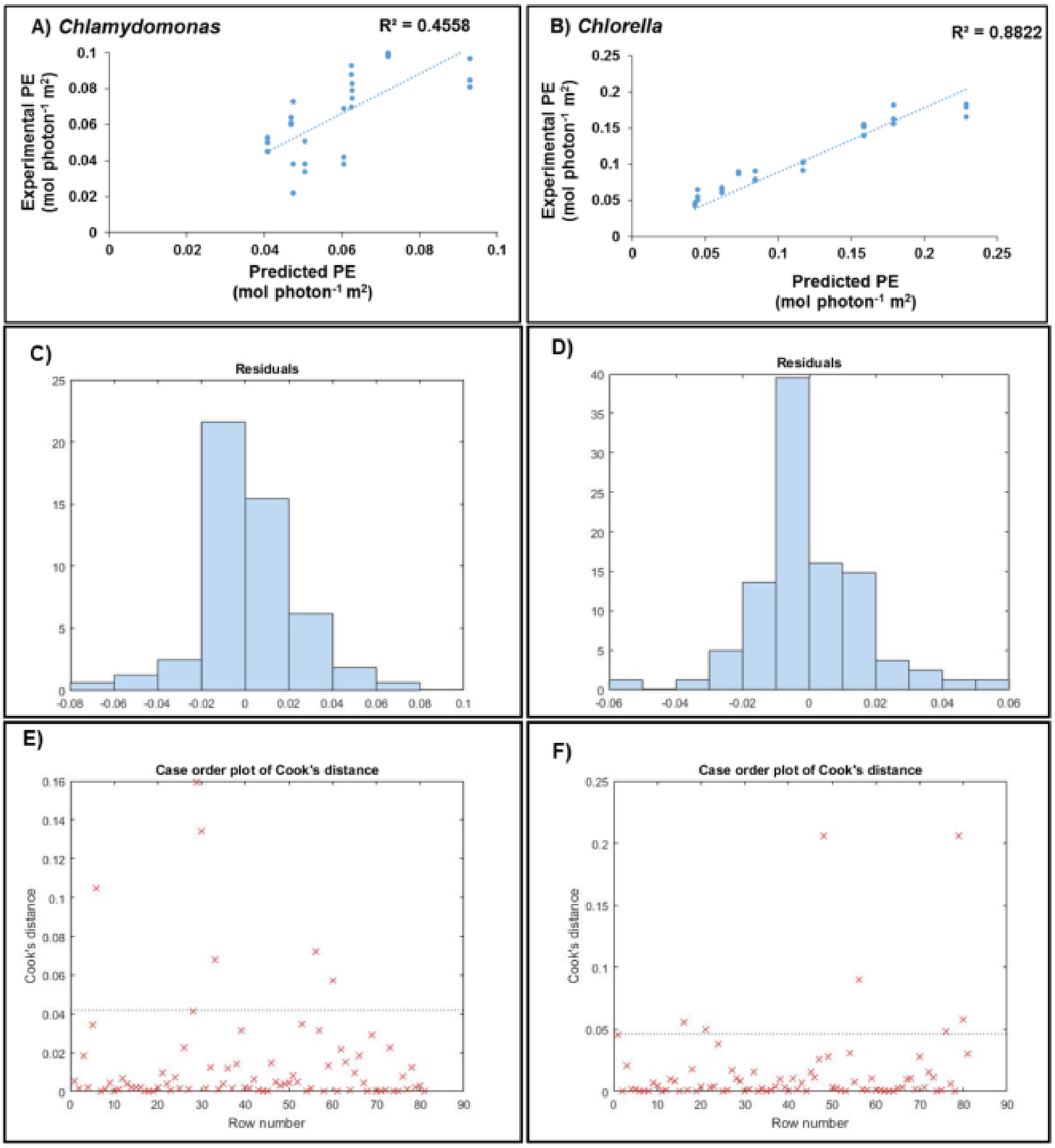

Supplementary Figure S5. Model validation using an independent data set. **A** and **B** display predicted versus experimental PE_{μ} data (to confirm sufficient goodness of fit), **C** and **D** display residuals plot (to confirm normal distribution) and **E** and **F** Cook's distance plot (to identify outliers) for *Chlamydomonas* and *Chlorella* respectively.

Supplementary Table S2 for Section 3.4

Supplementary Table S2. Factor coefficients obtained from analysis of variance (ANOVA) for NPQ and OD_{680}/OD_{750}. All data represent the mean of 3 replicates ± standard deviation.

* represents significant effects at p-value<0.05

	Coefficients from the quadratic non-linear model			
	NPQ		OD_{680}/OD_{750}	
	Chlamydomonas	***Chlorella***	***Chlamydomonas***	***Chlorella***
**D_f**	-0.0026	-0.0155*	0.2514*	0.1879*
**I_{max}**	0.0267*	-0.0134*	-0.2208*	0.0300*
**t_c**	0.0061	-0.0046	0.0106	-0.0674*
**D_f - I_{max}**	0.0006	-0.0098	0.0408*	0.02153
**D_f - t_c**	0.0056	-0.0008	-0.0162	0.0705*
**I_{max} - t_c**	0.0058	0.0003	0.0163	-0.0471*
**D_f^2**	-0.0313*	-0.0026	0.1206*	0.3359*
**I_{max}^2**	0.0141	-0.0101	0.0619*	-0.0143
**t_c^2**	0.0081	-0.0056	0.0078	-0.0093
**Intercept**	0.1729	0.0933	2.0436	1.734
**R^2**	0.49	0.37	0.89	0.85

Supplementary Table S3 for Section 5.1

Supplementary Table S3. Cell synchronisation procedure. The acclimation procedure for the light regime experiments based on the I_{max} considered. “Ticks” or “x” represent that particular step was or was not performed for the corresponding I_{max} experiment respectively. Note: For the higher intensity (I_{max} 1500), care was taken not to shock the cultures by subjecting them to a step-wise gradually increasing light regime.

Duration	Samples	Light regime	I_{max} 375	I_{max} 750	I_{max} 1500
~ 2-3 days	**Pre flasks** on shakers outside the TECAN	Constant overhead light ~100 µE	☑	☑	☑
~ 2-4 days	**Pre-flasks** in TECAN with overhead lights	Constant ~100 µE<br>L/D 16/8 h	☑	☒	☒
~ 2 days	**Pre-flasks** in TECAN with LED’s	TECAN LED’s 100µE constant light; L/D 16/8 h	☑	☒	☒
~ 24 hours	**Pre-flasks** in TECAN with LEDs	Constant 350 µE LEDs , L/D 16/8 h	☒	☒	☑
Pre flasks, 2 hours	**Pre-flasks** in TECAN	400 µE (1 h), 600 µE (1 h)	☒	☒	☑
1hour	Cultures adjusted to target starting OD_{750} ; Plated in **96 well plates**	TECAN LED’s at100µE constant light; L/D 16/8 h	☑	☑	☒
Start of experiment	96-well plates-light regime is set.	Considered ready for measurements as mentioned in the Methods section.<br>L/D 16/8 h	☑	☑	☒
Day 0, after plating	96 well plates- gradual light acclimation in progress for higher I_{max} experiment.	1 h 200 µE constant	☒	☒	☑
		2 h light cycles with I_{max} = 750 µE			
		First measurement as mentioned in the Methods section			
		8h dark period			
Day 1, AM, gradual acclimation	Acclimation of plates continue- before making the first dilution	First 4 h of light after dark period: Light regime with I_{max} = 750 µE	☒	☒	☑
		Next 6h – light regime with I_{max} = 1500 µE followed by dilution of samples			
Day 1, afternoon	Acclimation of plates continue- Post-dilution of plates.	2.5 h light regimes with I_{max} = 750 µE (for recovery after dilution)	☒	☒	☑
	Acclimation set.	2 h light regime with I_{max} = 1000 µE			
		1.5 h light regime with I_{max} = 1500 µE			
		8 h dark period			
Day 2, AM	Acclimation continues	10 h light regime with I_{max} = 1500 µE followed by dilution of cultures.	☒	☒	☑
Day 2, afternoon onwards up to Day 3 afternoon	Acclimation continues	Repeat same as Day 1 afternoon onwards	☒	☒	☑

Supplementary Figure S6 for Section 5.2

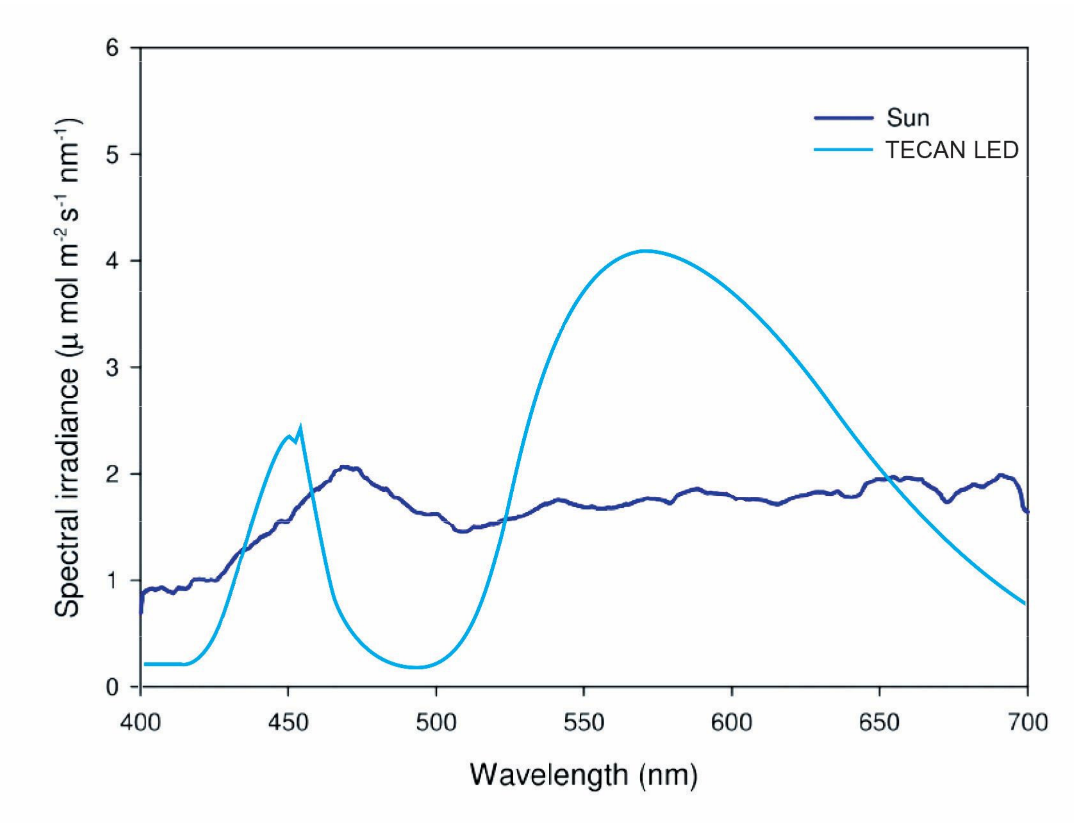

Supplementary Figure S6. Approximation of the spectral irradiance over the PAR spectrum (400-700nm) showing the difference between sunlight (dark blue line) and the warm white LEDs of the TECAN system (light blue line). Sun spectrum reproduced from Yarnold, J.[1]. TECAN spectrum reproduced from World of Thought Pty Ltd, Australia (www.worldofthought.com.au).

Reference

1 Yarnold, J. *Photosynthesis of microalgae in outdoor mass cultures and modelling its effects on biomass productivity for fuels, feeds and chemicals* PhD thesis, The University of Queensland, (2016).
